# High-Sensitivity Cardiac Troponin T and Cardiovascular Risk After Ischemic Stroke or Transient Ischemic Attack

**DOI:** 10.1016/j.jacadv.2024.101022

**Published:** 2024-06-04

**Authors:** Karin Willeit, Christian Boehme, Thomas Toell, Lena Tschiderer, Lisa Seekircher, Lukas Mayer-Suess, Silvia Komarek, Wilfried Lang, Andrea Griesmacher, Michael Knoflach, Johann Willeit, Stefan Kiechl, Peter Willeit

**Affiliations:** a Department of Neurology, Medical University of Innsbruck, Innsbruck, Austria; b Department of Medical Statistics, Informatics and Health Economics, Medical University of Innsbruck, Innsbruck, Austria; c VASCage, Centre of Clinical Stroke Research, Innsbruck, Austria; d Medical Faculty, Sigmund Freud Private University Vienna, Vienna, Austria; e Central Institute of Medical and Chemical Laboratory Diagnostics, University Hospital of Innsbruck, Innsbruck, Austria; f Department of Public Health and Primary Care, University of Cambridge, Cambridge, United Kingdom

**Keywords:** cardiovascular risk, ischemic stroke, secondary prevention, transient ischemic attack, troponin T

## Abstract

**Background:**

High-sensitivity cardiac troponin T (hs-cTnT) is associated with cardiovascular disease (CVD) risk in general and various high-risk populations.

**Objectives:**

The purpose of this study was to precisely characterize the association of hs-cTnT with CVD risk in patients following acute ischemic stroke or transient ischemic attack.

**Methods:**

We conducted post hoc analyses of data from the STROKE-CARD trial (NCT02156778), a pragmatic randomized controlled trial of a disease management program in patients with acute ischemic stroke or transient ischemic attack (ABCD^2^ score ≥3). We measured hs-cTnT on admission (Roche Elecsys, detection limit 5 ng/L) and quantified HRs for a composite CVD outcome (ie, stroke, myocardial infarction, CVD death) adjusted for age, sex, prior coronary heart disease, prior heart failure, diabetes, smoking, systolic blood pressure, and low- and high-density-lipoprotein cholesterol.

**Results:**

Among 1,687 patients (mean age, 69.3 ± 13.7 years; 40.7% female), hs-cTnT was detectable in 80.7%. Median hs-cTnT was 10 ng/L (IQR: 6-18 ng/L). Over a median follow-up of 12.1 months, 110 patients had a CVD event. The association of hs-cTnT level with CVD risk was of log-linear shape, with a multivariable-adjusted HR of 1.40 (95% CI: 1.15-1.70; *P* < 0.001) per 1-SD higher log-transformed hs-cTnT value. The strength of association was similar when further adjusted for other potential confounders and across clinically relevant subgroups. Corresponding outcome-specific HRs were 1.33 (95% CI: 1.06-1.68; *P* = 0.016) for stroke, 1.28 (95% CI: 0.69-2.37; *P* = 0.430) for myocardial infarction, 1.98 (95% CI: 1.43-2.73; *P* < 0.001) for CVD death, and 1.93 (95% CI: 1.54-2.41; *P* < 0.001) for all-cause death.

**Conclusions:**

High hs-cTnT is associated with increased CVD risk in ischemic stroke and transient ischemic attack patients.

Recurrent cardiovascular disease (CVD) events following ischemic stroke or transient ischemic attack (TIA) are a major medical challenge. Risk of subsequent stroke in stroke and TIA patients is particularly high within the first year after the event, with 1-year cumulative risk estimates ranging from 4.4% to 13.1%.^1^^,^^2^ Poststroke cardiac complications are also common, with a recent large population-based cohort study reporting a 4.5-fold increased risk of cardiac events in the year after ischemic stroke.^3^ Several risk scores incorporating demographic and clinical parameters have been developed to estimate risk of recurrent CVD events after ischemic stroke or TIA, but their accuracy in predicting an individual’s CVD risk is limited.^4^

Circulating biomarkers have been recognized increasingly as valuable tools to improve risk prediction by reflecting different pathological pathways. In this context, high-sensitivity cardiac troponin T (hs-cTnT) is particularly promising^5^ because it is a highly sensitive and specific marker of myocardial injury. In a literature-based meta-analysis involving 28 studies and 154,052 participants without prior CVD, we have previously shown that high-sensitivity cardiac troponin is detectable in 80.0% of individuals and that values even within the normal range are associated with CVD risk independent of conventional risk factors.^6^ Similarly, individual studies have shown associations with CVD risk in patients with coronary heart disease (CHD),^7^ heart failure,^8^ and atrial fibrillation (AF).^9^ In contrast, whether high-sensitivity cardiac troponins are also linked to elevated CVD risk in patients with cerebrovascular disease is less clear because prior studies reported inconsistent results, and most had limited statistical power or predominantly focused on TIA or stroke of mild to moderate severity.^10^, ^11^, ^12^, ^13^, ^14^

To address this uncertainty, we assessed the association of hs-cTnT levels with recurrent CVD events in the STROKE-CARD trial, a pragmatic trial of a disease management program, which included patients with a broad spectrum of disease severity spanning from moderate-risk TIA to major ischemic stroke.

## Methods

### Study population

The STROKE-CARD trial was a pragmatic open-label trial (NCT02156778) that randomly assigned patients to receive STROKE-CARD care or standard care.^15^ Its design has been described previously.^16^ In brief, between January 2014 and December 2017, 2,149 patients aged ≥18 years with an acute ischemic stroke (defined according to American Heart Association criteria)^17^ or TIA (ABCD^2^ score ≥3)^18^ were enrolled at 2 trial centers, that is, Innsbruck University Hospital and Hospital St. John’s of God Vienna. Exclusion criteria were malignancies or other severe diseases with an estimated life expectancy <1 year, drug addiction, severe alcohol abuse, residence outside the catchment area, or severe disability with low probability of successful rehabilitation (ie, modified Rankin Scale score of 5 at hospital discharge). In the control arm, patients were managed according to the standard stroke care protocol.^19^ In the intervention arm, patients received STROKE-CARD care, which included a 3-month outpatient appointment performed by a multidisciplinary stroke team and access to a web-based patient portal. Thereby, STROKE-CARD care aimed to target risk factor management, to systematically detect and treat poststroke complications, and to enhance patient education, counseling, and self-empowerment. The study was approved by the ethics committee of the Medical University of Innsbruck; each patient gave written informed consent.

The present post hoc analysis focused on patients enrolled at the Innsbruck University Hospital. This trial center had measured and recorded hs-cTnT as part of routine diagnostic work-up and enrolled the majority of STROKE-CARD trial participants (80.5%). Of 1,730 eligible participants, 1,687 (97.5%) had complete information on hs-cTnT and variables required for statistical adjustment and were therefore included in the present analysis. As shown in Supplemental Table 1, there were some differences between patients included and those not included in the present analysis in terms of type of qualifying event and etiology.

### Outcome definition and assessment

Consistent with the prespecified primary outcome definition of our trial,^15^ we defined the combined CVD endpoint as occurrence of nonfatal myocardial infarction (MI), nonfatal stroke, or CVD death between hospital discharge and the 12-month follow-up visit. We also quantified additional associations for different types of outcomes, including angina pectoris, AF, heart failure, and cause-specific mortality. All outcomes were adjudicated by a committee blinded to group assignment using standard diagnostic criteria.^17^^,^^20^, ^21^, ^22^, ^23^

### Laboratory methods

As part of clinical routine, blood samples were drawn: 1) once upon presentation to the emergency ward (median time since known time of symptoms onset: 3 hours 21 minutes [IQR: 1 hour 33 minutes-19 hours 20 minutes]; 80.2% within 24 hours); 2) once on the following day; and 3) additionally only if clinically indicated. The principal analysis of the present study focused on admission hs-cTnT (ie, the first value available upon hospital presentation), while a sensitivity analysis focused on hs-cTnT measured closest to hospital discharge. The distribution of time points at which hs-cTnT were measured, histograms and scatter plots of hs-cTnT values measured at admission vs closest to hospital discharge are provided in Supplemental Figure 1.

Blood samples were processed immediately. hs-cTnT levels were measured using a high-sensitivity electrochemiluminescence immunoassay (Elecsys 2010 system; Roche Diagnostics) with a limit of blank set at 3 ng/L, a limit of detection of 5 ng/L, an upper reference limit corresponding to the 99th percentile of a healthy reference population of 14 ng/L,^20^^,^^24^ and sex-specific upper reference limits of 10.0 and 14.5 ng/L in females and males, respectively.^24^ For the purpose of this analysis, 325 of 1,687 patients (19.3%) had a hs-cTnT value below the limit of detection and were assigned a value of 2.5 ng/L. Assays used to measure other parameters are described in the Supplemental Methods.

### Statistical analysis

Baseline patient characteristics were summarized using mean ± SD median (IQR), or counts (percentages), as appropriate. Values of hs-cTnT, N-terminal pro–B-type natriuretic peptide (NT-proBNP), and high-sensitivity C-reactive protein (hs-CRP) values had a skewed distribution and were log-transformed for further analysis. Cross-sectional associations of log-transformed hs-cTnT with other baseline characteristics were assessed using sex-specific correlation plots, partial correlation coefficients, and age- and sex-adjusted linear regression models.

We analyzed time-to-event data from hospital discharge to occurrence of the event of interest, death, or end of follow-up, whichever had occurred first. For patients who had attended the 12-month visit late (ie, >13 months after hospital discharge), time-to-event data were censored at 13 months. The proportional hazards assumption was tested using Schoenfeld residuals and was met. We estimated HRs using Cox regression models stratified by trial arm. To characterize shapes of associations with CVD risk, we calculated HRs across the groups of patients with undetectable hs-cTnT (reference group) and patients in the 4 quartiles of detectable hs-cTnT levels. In this analysis, we used floating absolute risks to calculate 95% CIs for all groups including the reference, thereby allowing head-to-head comparisons between effect sizes of any 2 of the groups.^25^ Furthermore, we calculated HRs of hs-cTnT per 1-SD higher log-transformed level, which corresponds to a 2.70-fold change on the original scale (ie, e^0.993^). We progressively adjusted HRs for age, sex, prior CHD, diabetes, smoking, systolic blood pressure, low- and high-density-lipoprotein cholesterol, and prior heart failure (“multivariable adjusted model”), and made further adjustment for log-transformed NT-proBNP, estimated glomerular filtration rate (eGFR), log-transformed hs-CRP, history of AF, type of qualifying event, and admission of National Institutes of Health Stroke Scale (NIHSS) score >5 in subsidiary analyses. Furthermore, we investigated effect modification by trial arm, type of qualifying event, stroke etiology, age, sex, AF, and prior CHD with formal tests of interaction. To assess the incremental value of hs-cTnT for CVD prediction, we calculated C-index changes^26^ and net reclassification improvements^27^ when adding hs-cTnT to 2 established risk scores in stroke/TIA patients,^4^ that is, the Stroke Prognosis Instrument-II Score and the Essen Stroke Risk Score. We quantified net reclassification across the 12-month risk categories <2.5%, 2.5%-<5%, 5%-<7.5%, and ≥7.5%, corresponding approximately to quartiles of predicted risk.

Analyses were performed with Stata 16.1 MP and used 2-sided statistical tests and a significance level of *P* < 0.05 (apart from a Bonferroni-corrected significance level of *P* < 0.0056 in the subgroup analysis).

## Results

### High-sensitvity cardiac troponin and other baseline characteristics

hs-cTnT was detectable in 1,362 (80.7%) participants and was above the general 99th percentile upper reference limit in 592 (35.1%) participants and above the sex-specific 99th percentile upper reference limit in 689 (40.8%) participants. Median hs-cTnT concentration was 10 ng/L (IQR: 6-18 ng/L), mean age was 69.3 ± 13.7 years, and 687 patients (40.7%) were female (Table 1). As the event qualifying patients for trial inclusion, 1,398 (82.9%) had an ischemic stroke and 289 (17.1%) a TIA.

**Table 1:** Baseline Characteristics of Study Participants and Their Cross-Sectional Associations With Admission hs-cTnT (N = 1,687)

		Age and Sex-Adjusted % Difference (95% CI) in hs-cTnT Per 1-SD Increase or Compared to Reference^a^	*P* Value
Type of qualifying event			
Transient ischemic attack	289 (17.1%)	[Reference]	[Reference]
Ischemic stroke	1,398 (82.9%)	19% (9-27)	<0.001
TOAST classification			
Large-artery atherosclerosis	336 (19.9%)	[Reference]	[Reference]
Cardiac embolism	437 (25.9%)	34% (19-51)	<0.0001
Small-artery occlusion	357 (21.2%)	−14% (−24 to −3)	0.016
Uncommon causes	71 (4.2%)	16% (−7 to 44)	0.193
Undetermined causes	486 (28.8%)	−8% (−18 to 3)	0.157
Admission NIHSS score >5	383 (22.7%)	30% (19-43)	<0.0001
Discharge modified Rankin Scale^b^			
No symptoms	537 (31.8%)	[Reference]	[Reference]
No significant disability	348 (20.6%)	16% (4-29)	0.010
Slight disability	462 (27.4%)	28% (15-42)	<0.0001
Moderate disability	234 (13.9%)	71% (50-95)	<0.0001
Moderately severe disability	106 (6.3%)	80% (52-114)	<0.0001
Age (y)	69.3 ± 13.7	69% (62-76)	<0.0001
Female	687 (40.7%)	−21% (−27 to −14)	<0.0001
Prior coronary heart disease	254 (15.1%)	56% (39-74)	<0.0001
Atrial fibrillation	424 (25.1%)	52% (38-68)	<0.0001
Prior heart failure	155 (9.2%)	91% (66-119)	<0.0001
Diabetes mellitus	314 (18.6%)	40% (26-55)	<0.0001
Smoking	363 (21.5%)	−7% (−16 to 3)	0.187
History of hypertension	1,367 (81.0%)	21% (8-35)	0.001
Systolic blood pressure (mm Hg)	135 ± 18	−2% (−6 to 2)	0.399
Diastolic blood pressure (mm Hg)	77.8 ± 11.7	−8% (−12 to −4)	<0.0001
LDL cholesterol (mmol/L)	2.95 ± 0.99	−13% (−17 to −10)	<0.0001
HDL cholesterol (mmol/L)	1.29 ± 0.41	−9% (−12 to −5)	<0.0001
NT-proBNP (ng/L)^c^^,^^d^	269 (103-883)	57% (50-64)	<0.0001
hs-CRP (mg/L)^d^	3.1 (1.3-8.3)	19% (15-24)	<0.0001
eGFR (mL/min/1.73 m^2^)	70.8 ± 19.3	−24% (−27 to −20)	<0.0001

Values are n (%), mean ± SD, or median (IQR) unless otherwise indicated.

eGFR = estimated glomerular filtration rate; HDL = high-density lipoprotein; hs-CRP = high-sensitivity C-reactive protein; hs-cTnT = high-sensitivity cardiac troponin T; LDL = low-density lipoprotein; NIHSS = National Institutes of Health Stroke Scale; NT-proBNP = N-terminal pro-B-type natriuretic peptide; TIA = transient ischemic attack.

a Percent difference in hs-cTnT per 1-SD higher value of the row variable or, for categorical variables, compared to reference category. hs-cTnT values were log-transformed for analyses.

b Due to trial inclusion criteria, there was no patient with severe disability at discharge.

c NT-proBNP measurements were available in 1,547 patients.

d Because distributions of NT-proBNP and hs-CRP were skewed, % differences in hs-cTnT were calculated per SD in log-transformed NT-proBNP and hs-CRP, corresponding to 4.54-fold and 3.97-fold differences, respectively.

**Figure 1:**
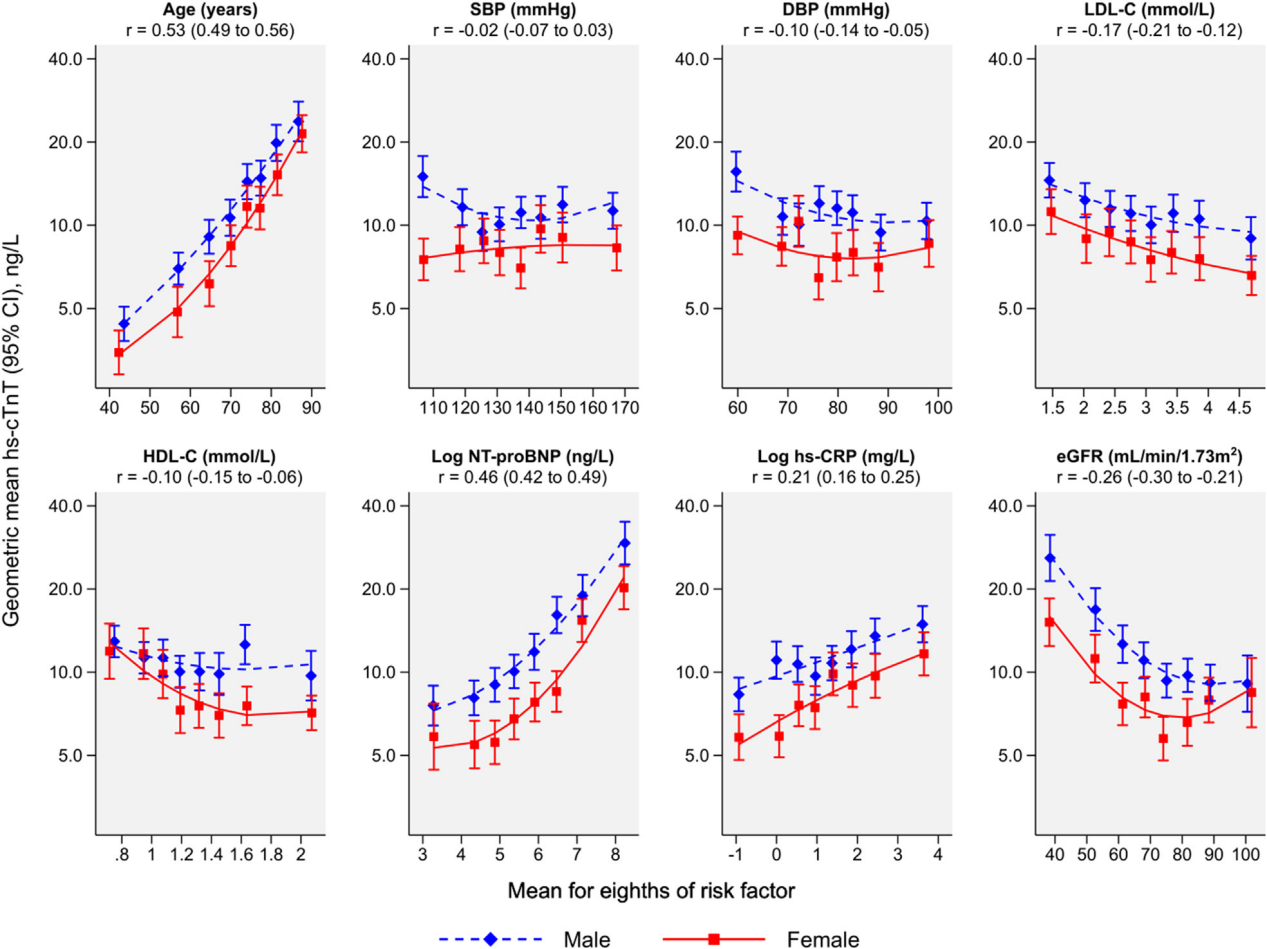
**Cross-sectional Associations Between hs-cTnT and Continuous CVD Risk Factors** The geometric mean hs-cTnT level was adjusted to age 70 years. Partial correlation coefficients (r) were calculated for males and females combined and were adjusted for age and sex. DBP = diastolic blood pressure; eGFR = estimated glomerular filtration rate; HDL-C = high-density-lipoprotein cholesterol; hs-CRP = high-sensitivity C-reactive protein; hs-cTnT = high-sensitivity cardiac troponin T; LDL-C = low-density-lipoprotein cholesterol; NT-proBNP = N-terminal pro-B-type natriuretic peptide; SBP = systolic blood pressure.

In cross-sectional analyses, higher hs-cTnT levels were associated with older age, a higher NT-proBNP, hs-CRP, and discharge modified Rankin Scale score, with ischemic stroke as the qualifying event, with prior CHD, prior heart failure, diabetes, AF, history of hypertension, a NIHSS score >5 at admission, and cardioembolic etiology of the qualifying event (Table 1). Levels of hs-cTnT were inversely associated with female sex, diastolic blood pressure, low- and high-density-lipoprotein cholesterol, and eGFR. Figure 1 shows cross-sectional associations with continuous CVD risk factors separately for males and females.

### Association with incident cardiovascular events

**Figure 2:**
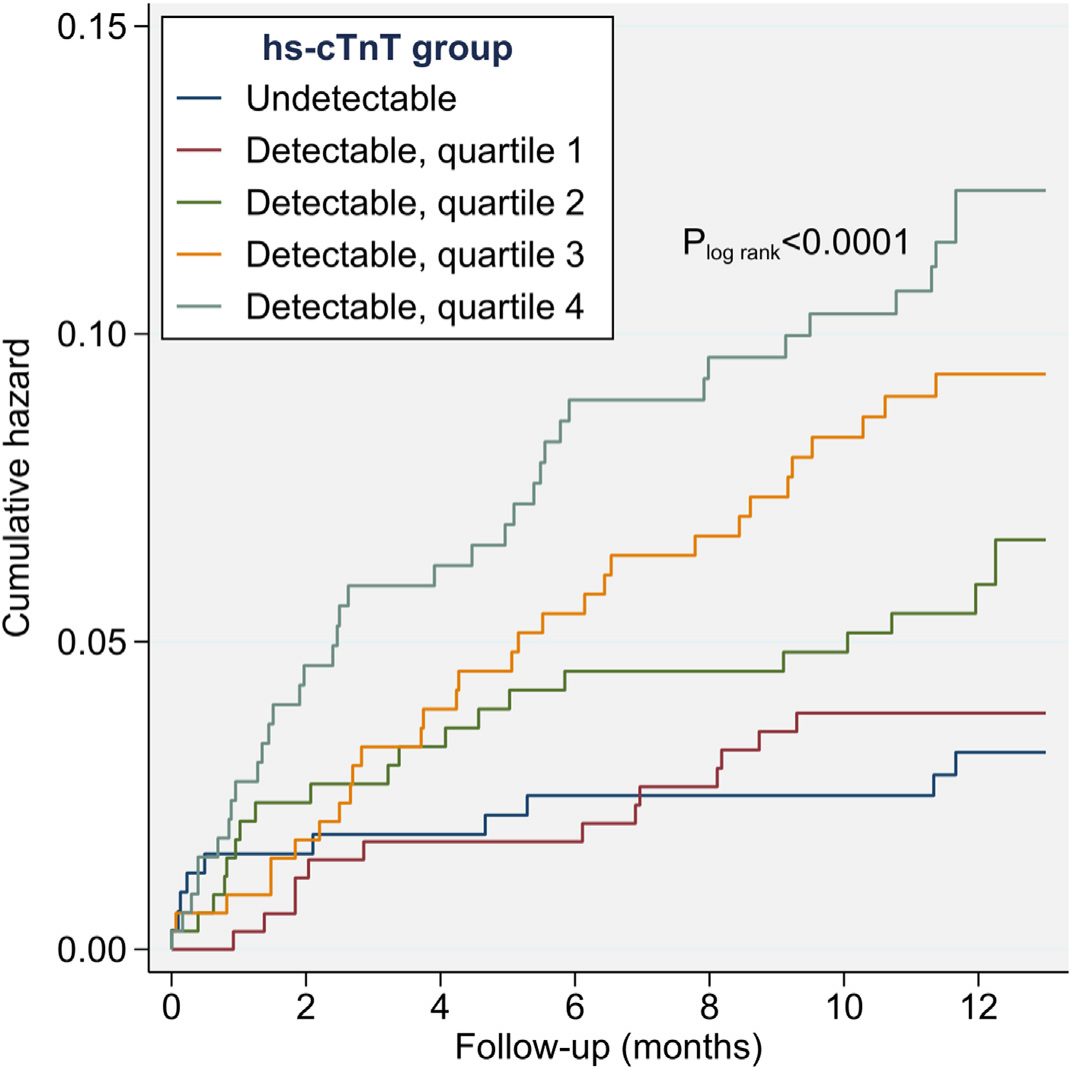
**Cumulative Hazard for the Combined CVD Endpoint by Undetectable and Quartiles of Detectable hs-cTnT Values** Minimum and maximum values (ng/L) of detectable hs-cTnT were 5.0 to 8.2 in quartile 1, 8.3 to 12.4 in quartile 2, 12.5 to 20.9 in quartile 3, and ≥21.0 in quartile 4. hs-cTnT = high-sensitivity cardiac troponin T.

Over a median follow-up of 12.1 months (5th-95th percentile: 4.8-13.0 months), 110 CVD events occurred, including 78 nonfatal strokes (72 ischemic, 6 hemorrhagic), 13 nonfatal myocardial infarctions, and 19 vascular deaths. The incidence rate was 68.3 per 1,000 person-years (95% CI: 56.7-82.3) overall and increased with higher hs-cTnT category from 31.2 (95% CI: 16.8-57.9) in patients with undetectable hs-cTnT, to 38.2 (95% CI: 22.2-65.8) in the first, 70.0 (95% CI: 39.3-94.5) in the second, 93.1 (95% CI: 65.1-133.2) in the third, and 123.8 (95% CI: 89.7-170.8) in the fourth quartile of detectable hs-cTnT (log-rank *P* < 0.0001) (Figure 2).

**Figure 3:**
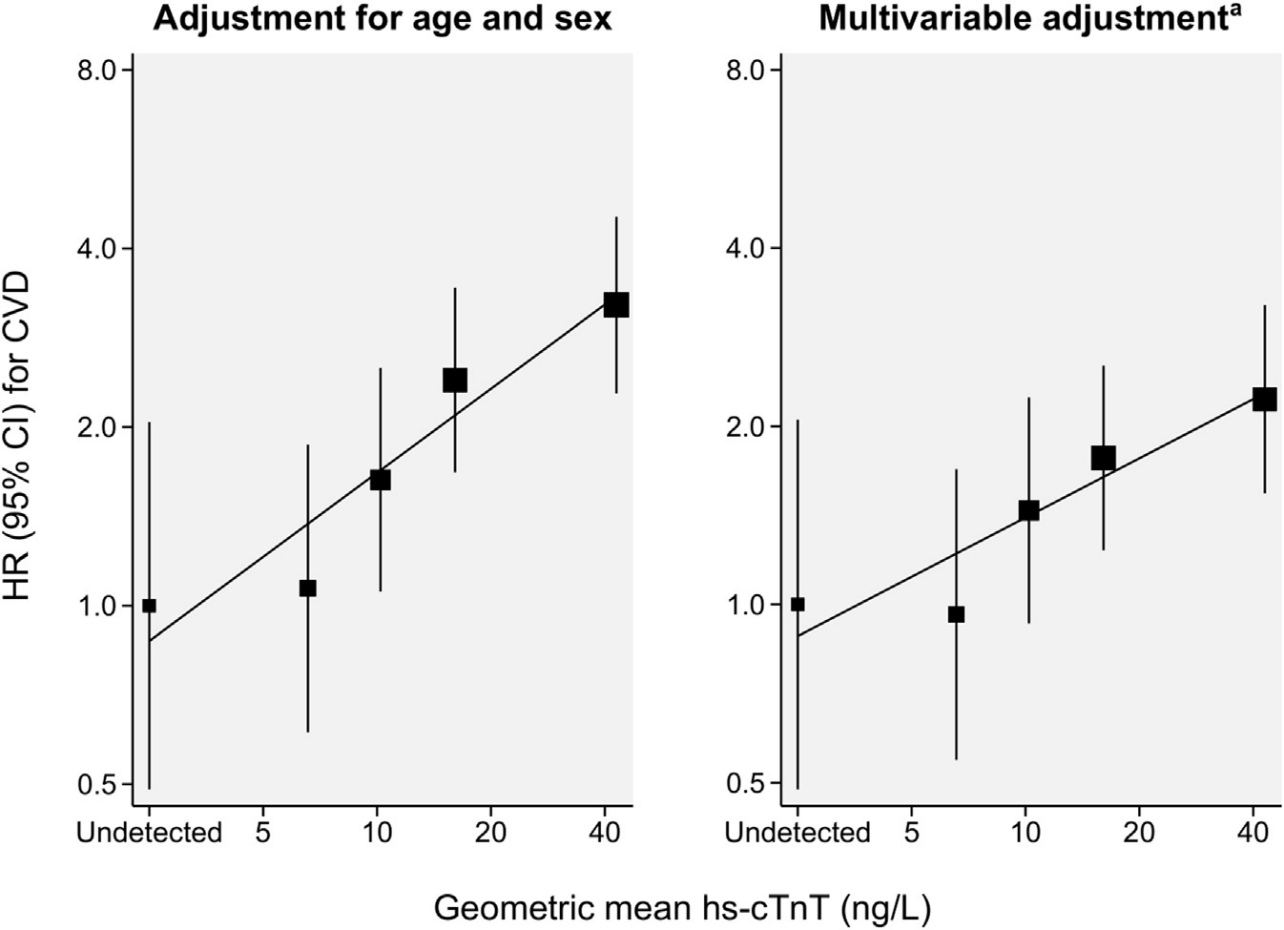
**Shapes of Association of Hs-cTnT With the Risk of the Combined CVD Endpoint Across the Groups of Undetectable and Quartiles of Detectable hs-cTnT Values** Sizes of boxes are proportional to the inverse of the variance of the HRs. The group with undetectable hs-cTnT values was used as the reference group. Floating absolute risks were used to calculate 95% CIs for all groups including the reference, thereby allowing head-to-head comparisons between effect sizes of any 2 of the groups. ^a^Adjusted for age, sex, prior coronary heart disease, prior heart failure, diabetes, smoking, systolic blood pressure, low- and high-density-lipoprotein cholesterol. CVD = cardiovascular disease; hs-cTnT = high-sensitivity cardiac troponin T.

**Figure 4:**
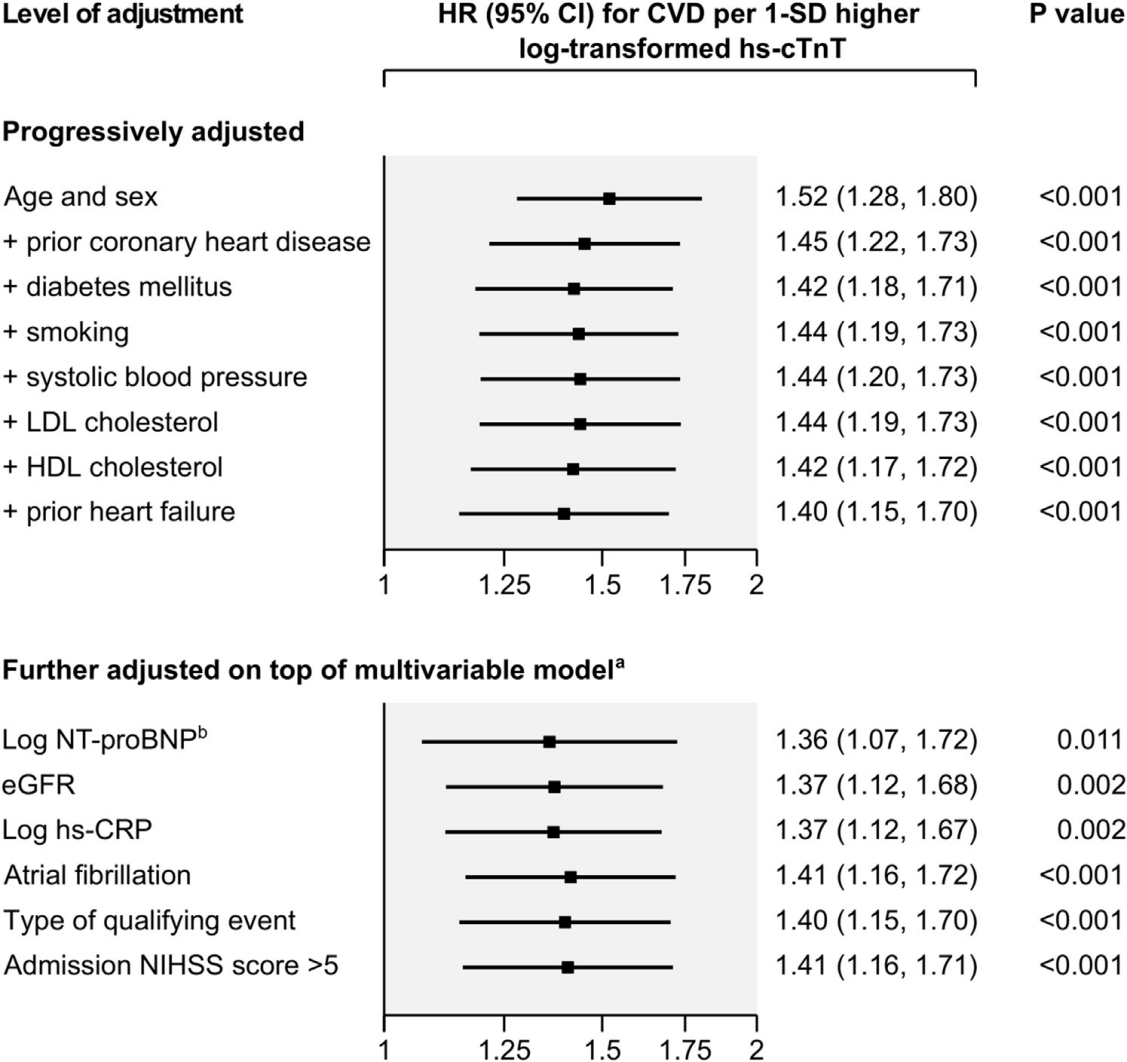
**Association of hs-cTnT Levels With the Risk of the Combined CVD Endpoint According to Different Levels of Adjustment (N = 1,687, 110 CVD Events)** ^a^Further adjusted models employed adjustment for each of the presented variables in turn, on top of the multivariable model including age, sex, prior coronary heart disease, prior heart failure, diabetes, smoking, systolic blood pressure, and low- and high-density-lipoprotein cholesterol. ^b^This analysis involves 1,547 patients and 99 CVD events; for head-to-head comparison, the multivariable-adjusted HR in the same subset of patients was 1.36 (95% CI: 1.10-1.68; *P* = 0.004). CVD = cardiovascular disease; eGFR = estimated glomerular filtration rate; HDL = high-density lipoprotein; hs-CRP = high-sensitivity C-reactive protein; hs-cTnT = high-sensitivity cardiac troponin T; LDL = low-density lipoprotein; NIHSS = National Institutes of Health Stroke Scale; NT-proBNP = N-terminal pro-B-type natriuretic peptide.

In Cox regression analysis, the association of hs-cTnT and risk of CVD was positive and of log-linear shape (Figure 3). A 1-SD higher log-transformed level of hs-cTnT was associated with a HR for incident CVD of 1.52 (95% CI: 1.28-1.80; *P* < 0.001) in a model adjusted for age and sex, and 1.40 (95% CI: 1.15-1.70; *P* < 0.001) in the multivariable adjusted model (Figure 4). Further adjustment for NT-proBNP, eGFR, hs-CRP, AF, type of qualifying event, and NIHSS score >5 at admission did not materially change the strength of association (Figure 4). In a head-to-head comparison, the strength of association of hs-cTnT with CVD risk was comparable to having lower high-density-lipoprotein cholesterol, while associations of other cardiovascular risk markers including hs-CRP and NT-proBNP were nonsignificant with HR point estimates closer to the null (Supplemental Figure 2).

In sensitivity analyses, results were almost identical when the multivariable-adjusted analysis: 1) omitted 325 individuals with undetectable hs-cTnT (1.44, 95% CI: 1.16-1.78; *P* < 0.001); 2) omitted 10 individuals that had a MI during hospitalization for the qualifying event (1.41, 95% CI: 1.15-1.74; *P* < 0.001); 3) used hs-cTnT values measured closest in time to hospital discharge rather than admission hs-cTnT (1.39, 95% CI: 1.13-1.71; *P* = 0.001); 4) was adjusted for history of hypertension rather than baseline systolic blood pressure (1.39, 95% CI: 1.14-1.69; *P* < 0.001); 5) took into account competing risks with non-CVD mortality (1.40, 95% CI: 1.17-1.67; *P* < 0.001); and 6) restricted to the 1,639 patients with hs-cTnT measured within a day of admission (1.40, 95% CI: 1.15-1.71; *P* < 0.001). Furthermore, associations were broadly similar across relevant subgroups including trial arm, type of qualifying event, cardioembolic etiology of the qualifying event, age, sex, AF, prior CHD, prior heart failure, and severity of the qualifying event (NIHSS >5 vs ≤5), indicated by nonsignificant tests of interaction (all *P* < 0.0071) (Supplemental Figure 3).

**Figure 5:**
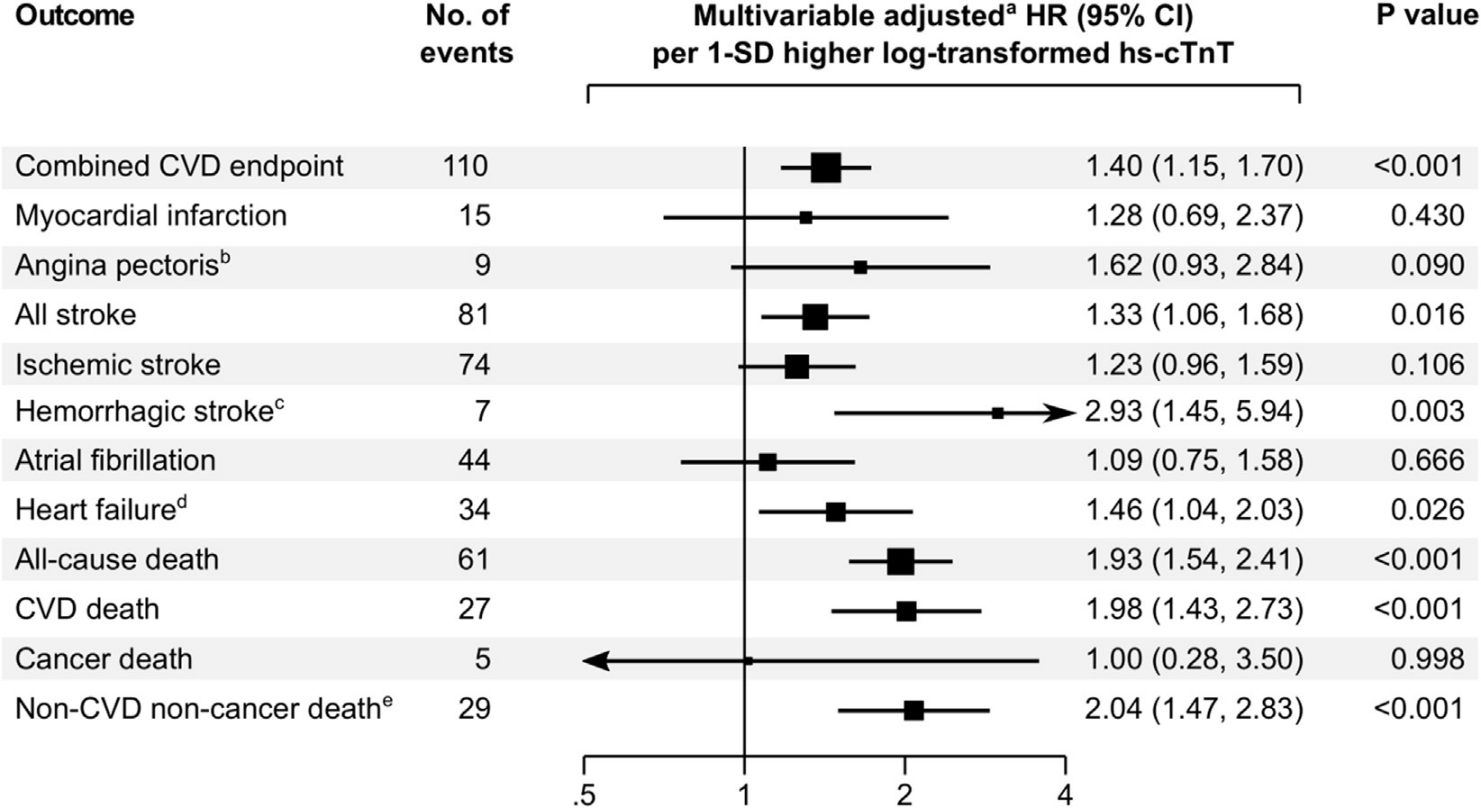
**Multivariable-AdjustedAssociation of hs-cTnT With Individual Cardiovascular and Mortality Outcomes** ^a^Adjusted for age, sex, prior coronary heart disease, prior heart failure, diabetes, smoking, systolic blood pressure, and low- and high-density-lipoprotein cholesterol. ^b^Excluded 61 participants with prior angina pectoris at baseline. ^c^Comprises 4 cases of intracerebral hemorrhage and 3 cases of subarachnoid hemorrhage. ^d^Excluded 155 participants with prior heart failure at baseline. ^e^Comprises deaths due to pneumonia (n = 8), sepsis (n = 7), frailty (n = 6), renal disease (n = 3), chronic obstructive pulmonary disease (n = 1), severe nose bleeding with massive reduction in hemoglobin (n = 1), traumatic brain injury (n = 1), peritonitis (n = 1), and suicide (n = 1). CVD = cardiovascular disease; hs-cTnT = high-sensitivity cardiac troponin T.

When examining the association separately with individual cardiovascular outcomes, hs-cTnT levels were significantly associated with a higher risk of all stroke, hemorrhagic stroke, and heart failure, whereas no significant association was observed for MI, angina pectoris, ischemic stroke, and AF. For mortality outcomes, hs-cTnT was significantly associated with all-cause death, CVD death, and non-CVD non-cancer death, but not with cancer death (Figure 5).

### Added value for CVD prediction

Information on hs-cTnT changed the C-index from 0.614 to 0.654, corresponding to an improvement by 0.040 (95% CI: 0.011-0.070; *P* = 0.008) when added to the Stroke Prognosis Instrument-II and from 0.592 to 0.656, corresponding to an improvement by 0.065 (95% CI: 0.031-0.098; *P* < 0.001) when added to the Essen Stroke Risk Score. The corresponding net reclassification improvements across 12-month predicted risk categories were 0.212 (95% CI: 0.105-0.320) for addition to the Stroke Prognosis Instrument-II and 0.228 (95% CI: 0.107-0.348) for addition to the Essen Stroke Risk Score (both *P* < 0.001).

## Discussion

In this study of 1,687 patients from the STROKE-CARD trial, we showed that hs-cTnT is significantly associated with incident CVD. The association was of a positive log-linear shape and of considerable strength, independent of conventional cardiovascular risk factors including prior CHD and heart failure, persistent after further adjustment for NT-proBNP, eGFR, hs-CRP, AF, type of qualifying event, and NIHSS score >5 at admission, and consistent across clinically relevant subgroups (Central Illustration). Among the individual CVD outcomes tested, we found no association between higher hs-cTnT levels and risk of MI, angina pectoris, ischemic stroke, and AF but a significant association with all stroke, hemorrhagic stroke, and heart failure. Higher levels of hs-cTnT were associated with increased risk of death, not just from CVD, but also from non-CVD non-cancer causes. Finally, we observed significant improvements in CVD prediction metrics when hs-cTnT was assessed on top of different clinical risk scores developed for use in this patient population.

**Central Illustration:**
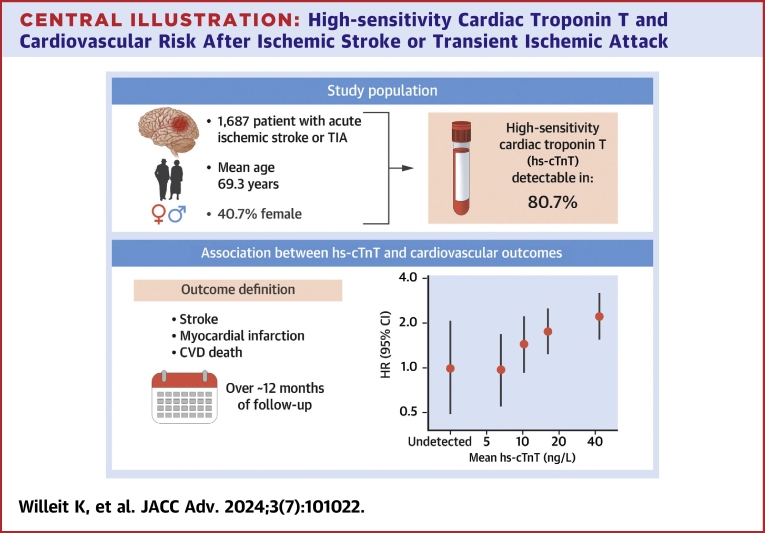
**High-sensitivity Cardiac Troponin T and Cardiovascular Risk After Ischemic Stroke or Transient Ischemic Attack** hs-cTnT = high-sensitivity cardiac troponin T; TIA = transient ischemic attack.

### Associations with cardiovascular and mortality outcomes

To date, only 5 studies have previously reported on associations between cardiac troponin concentration and occurrence of CVD events in patients after TIA or ischemic stroke.^10^, ^11^, ^12^, ^13^, ^14^ In line with our study, the INSPIRE-TMS study involving 889 patients with TIA or minor stroke found that hs-cTnT levels >14 ng/L were significantly associated with 1.63-fold risk of CVD over a mean follow-up of 3.2 years.^10^ Furthermore, the PROSCIS-B study reported a significant positive dose-response relationship across quartiles of hs-cTnT with a composite outcome of recurrent stroke, MI, and all-cause mortality in 562 patients after mild to moderate stroke.^11^ Likewise, elevated cardiac troponin I levels were associated with higher CVD risk in a Korean cohort of 1,092 patients with acute ischemic stroke.^14^ In contrast, 2 other studies suggested no^13^ or a possible^12^ association between hs-cTnT and incident CVD but were conducted in selected stroke populations and had limited statistical power. Our study extends these previous studies by covering a broad spectrum of patients spanning from moderate-risk TIA to severe ischemic stroke independent of underlying etiology and by providing more detailed analyses, including the shape of association, different adjustments, and separate analyses for individual CVD and mortality outcomes.

Of the individual CVD outcomes we investigated, associations of hs-cTnT were significant with the risk of stroke and heart failure. Our study thereby confirms previous reports of a positive association of hs-cTnT with all stroke from studies conducted in the general population and in patients with AF^6^^,^^28^ and lends support that this association is particularly strong for the hemorrhagic stroke subtype. The latter has been previously observed by the BiomarCaRE project, a pooled analysis of 9 prospective European community-dwelling cohorts.^29^ Possible mechanisms driving this association may include shared cardiovascular risk factors like hypertension;^30^ however, in our study, the association between hs-cTnT and incident hemorrhagic stroke persisted when adjusting for hypertension. For incident heart failure, our present study yielded an association of similar strength as in general population studies and patients with pre-existing CVD other than stroke.^31^ In contrast, for incident MI, we found no significant association possibly due to the low number of MI events (n = 15) and the resultant limited statistical power. In a literature-based meta-analysis involving 83,950 participants and 7,061 CVD events,^6^ there were marked differences between the included studies in the strengths of association between cardiac troponins and CHD risk (*I*^2^ = 77.6%), but—when pooled together—the association was positive and highly significant.

In our study, a higher level of hs-cTnT was also linked to an elevated risk of all-cause death, which is already well established in stroke patients.^32^, ^33^, ^34^ In line with the INSPIRE-TMS study,^10^ we now extend this finding to death from CVD, which in our study appeared to be a major contributor to the overall significant association between hs-cTnT and the combined CVD endpoint. We also found a significant association with non-CVD non-cancer death which mainly comprised death due to pneumonia, sepsis, and frailty. In this context, hs-cTnT may be considered a nonspecific biomarker, indicating the severity of co-existing cardiovascular and noncardiovascular comorbidities.^35^

### Biological mechanisms

Several underlying mechanisms may explain the association between hs-cTnT and CVD. hs-cTnT, a specific and sensitive biomarker of myocardial injury, strongly correlates with the presence of subclinical CHD or other cardiac structural and functional abnormalities such as AF, left ventricular hypertrophy, and heart failure, all of which lead to adverse cardiovascular outcomes.^32^^,^^36^^,^^37^ Asymptomatic coronary artery stenosis of >50% was found in 25% of patients with ischemic stroke^38^ and has been associated with a ≈5-fold increased risk of major vascular events.^39^ As studies have shown that cardiac troponins independently predict the detection of previously unknown AF in ischemic stroke patients,^40^^,^^41^ the association between elevated hs-cTnT and CVD may also be driven by the presence of undetected silent AF causing a high risk of ischemic stroke by cardiac embolism, heart failure, and death. As an alternative mechanism, the so-called “stroke-heart syndrome” may evolve as a direct consequence of stroke in the first days contributing to cardiac troponin elevation and short- and long-term unfavorable outcome.^37^ Furthermore, elevated hs-cTnT may reflect shared vascular risk factors that simultaneously affect the heart and brain, especially through small vessel disease, resulting in a poor prognosis.^30^ While we were able to adjust for several conventional and emerging risk factors (including markers of renal function and inflammation) and show independent associations, we cannot exclude the possibility of residual confounding.

### Risk prediction and clinical implications

A distinguishing feature of our study is that we rigorously evaluated the added value of hs-cTnT for CVD prediction and observed substantial improvements in the metrics of risk discrimination and reclassification in a scenario where the hs-cTnT level is assessed on top of established risk scores.^4^ This suggests that hs-cTnT may be useful to risk-stratify patients with acute ischemic stroke or TIA and may also help enhance efficiency of future clinical trials by helping enrich trials with patients with higher CVD event rates.^42^ Finally, it is noteworthy that hs-cTnT was associated with CVD risk in patients assigned to a comprehensive poststroke disease management program, albeit with a somewhat smaller HR than in patients receiving standard care (Supplemental Figure 3). This suggests that hs-cTnT confers residual CVD risk even in patients receiving intensified poststroke care and that this group may benefit from additional targeted preventive measures and a tailored cardiac workup.

### Study strengths and limitations

Our study has several strengths including its prospective design, inclusion of patients with a broad spectrum of disease severities ranging from moderate-risk TIA to major ischemic stroke, inclusion of all etiological subtypes of ischemic stroke, and a complete, standardized, and independent outcome assessment.

Our study also has limitations. We cannot exclude temporary elevation of hs-cTnT in some patients due to myocardial injury induced by the stroke itself (“stroke-heart syndrome”), however such elevations are known to also contribute to an unfavorable outcome in the long run.^37^ Furthermore, it is unlikely that elevations due to acute cardiac complications during hospitalization for the qualifying event may have impacted our overall findings, given that the strength of association remained virtually identical in sensitivity analyses omitting these patients (n = 10). Finally, findings were also similar in sensitivity analyses that used hs-cTnT-measured closest to discharge rather than at admission.

## Conclusions

Elevated hs-cTnT concentration is associated with an increased risk of CVD outcomes in patients after TIA or ischemic stroke. Our study adds to the growing evidence that hs-cTnT may well assist in improving CVD risk prediction. Clinical trials are warranted to test whether additional targeted preventive measures can help reduce risk in patients with high hs-cTnT.

## Funding support and author disclosure

The STROKE-CARD trial was financially supported by the University Hospital (Tirol Kliniken), Tyrolean Health Insurance Company (TGKK), the Tyrol Health Care Funds (TGF), and unrestricted research grants from Boehringer Ingelheim, Bayer Healthcare, Nstim Services, and Sanofi. Komarek, Lang, Knoflach, and Kiechl were supported by VASCage-Research Centre on Clinical Stroke Research. VASCage is a COMET Centre within the Competence Centers for Excellent Technologies (COMET) program and funded by the Federal Ministry for Climate Action, Environment, Energy, Mobility, Innovation and Technology, the Federal Ministry of Labor and Economy, and the federal states of Tyrol, Salzburg, and Vienna. COMET is managed by the Austrian Research Promotion Agency (Österreichische Forschungsförderungsgesellschaft). FFG Project number: 898252. Prof P. Willeit reports personal fees from Novartis Pharmaceuticals outside the submitted work. All other authors have reported that they have no relationships relevant to the contents of this paper to disclose.

Perspectives **COMPETENCY IN MEDICAL KNOWLEDGE:** This study suggests a graded association between elevated high-sensitivity cardiac troponin T concentration and cardiovascular risk in patients after ischemic stroke or transient ischemic attack.

**TRANSLATIONAL OUTLOOK:** Future research is needed to clarify whether patients with elevated high-sensitivity cardiac troponin T benefit from additional targeted preventive measures, including a tailored cardiac workup.
