# Supplementary material for: High-Sensitivity Cardiac Troponin T and Cardiovascular Risk After Ischemic Stroke or Transient Ischemic Attack
Source: JACC Adv. 2024 Jun 4;3(7):101022. doi: 10.1016/j.jacadv.2024.101022 (PMC11312770; doi:10.1016/j.jacadv.2024.101022)
Supplement: Supplemental methods, figures and table [file mmc1.pdf]

Supplemental Appendix

High-Sensitivity Cardiac Troponin T and Cardiovascular Risk after Ischemic Stroke or Transient Ischemic Attack

Willeit K et al.

SUPPLEMENTAL METHODS

CRP (CRP4, Tina-quant CRP IV), LDL (LDLC3, LDL-Cholesterol Gen.3) and HDL cholesterol (HDLC4, HDL-Cholesterol Gen.4), and creatinine (CREP2 Creatinine plus ver.2) levels were assayed using cobas c systems (Roche Diagnostics). NT-proBNP (Elecsys proBNP II) levels were measured by electrochemiluminescence immunoassay on cobas e immunoassay analyzer (Roche Diagnostics). eGFR was calculated using the 4-variable Modification of Diet in Renal Disease (MDRD) Study estimating equation[1].

REFERENCE

1. Levey AS, Coresh J, Greene T, et al. Using standardized serum creatinine values in the modification of diet in renal disease study equation for estimating glomerular filtration rate. Ann Intern Med. 2006;145:247–254.

SUPPLEMENTAL TABLES

SUPPLEMENTAL TABLE 1 Comparison of characteristics of patients included in the present analysis and those not included.

Variable	Patients included in the present analysis (n=1687)	Patients not included in the present analysis (n=43)	P value[a]
Ischemic stroke vs. TIA as qualifying event	1398 (82.9%)	26 (60.5%)	<0.001
TOAST classification			<0.001
Large-artery atherosclerosis	336 (19.9%)	24 (55.8%)	
Cardiac embolism	437 (25.9%)	6 (14.0%)	
Small-artery occlusion	357 (21.2%)	4 (9.3%)	
Uncommon causes	71 (4.2%)	5 (11.6%)	
Undetermined causes	486 (28.8%)	4 (9.3%)	
Admission NIHSS score >5	383 (22.7%)	5 (11.6%)	0.086
Discharge modified Rankin Scale			0.16
No symptoms	537 (31.8%)	19 (44.2%)	
No significant disability	348 (20.6%)	8 (18.6%)	
Slight disability	462 (27.4%)	8 (18.6%)	
Moderate disability	234 (13.9%)	3 (7.0%)	
Moderately severe disability	106 (6.3%)	5 (11.6%)	
Age (years)	69.3±13.7	70.8±11.8	0.48
Female sex	687 (40.7%)	12 (27.9%)	0.091

[a]P values were calculated using χ^2-tests or two-sample independent t-tests. NIHSS = National Institutes of Health Stroke Scale; TIA = transient ischemic attack.

SUPPLEMENTAL FIGURES

SUPPLEMENTAL FIGURE 1 Distribution of time points of intrahospital hs-cTnT measurements (upper graph) and marginal scatter plot of hs-cTnT values measured at admission vs. hs-cTnT values measured closest to discharge (lower graph).

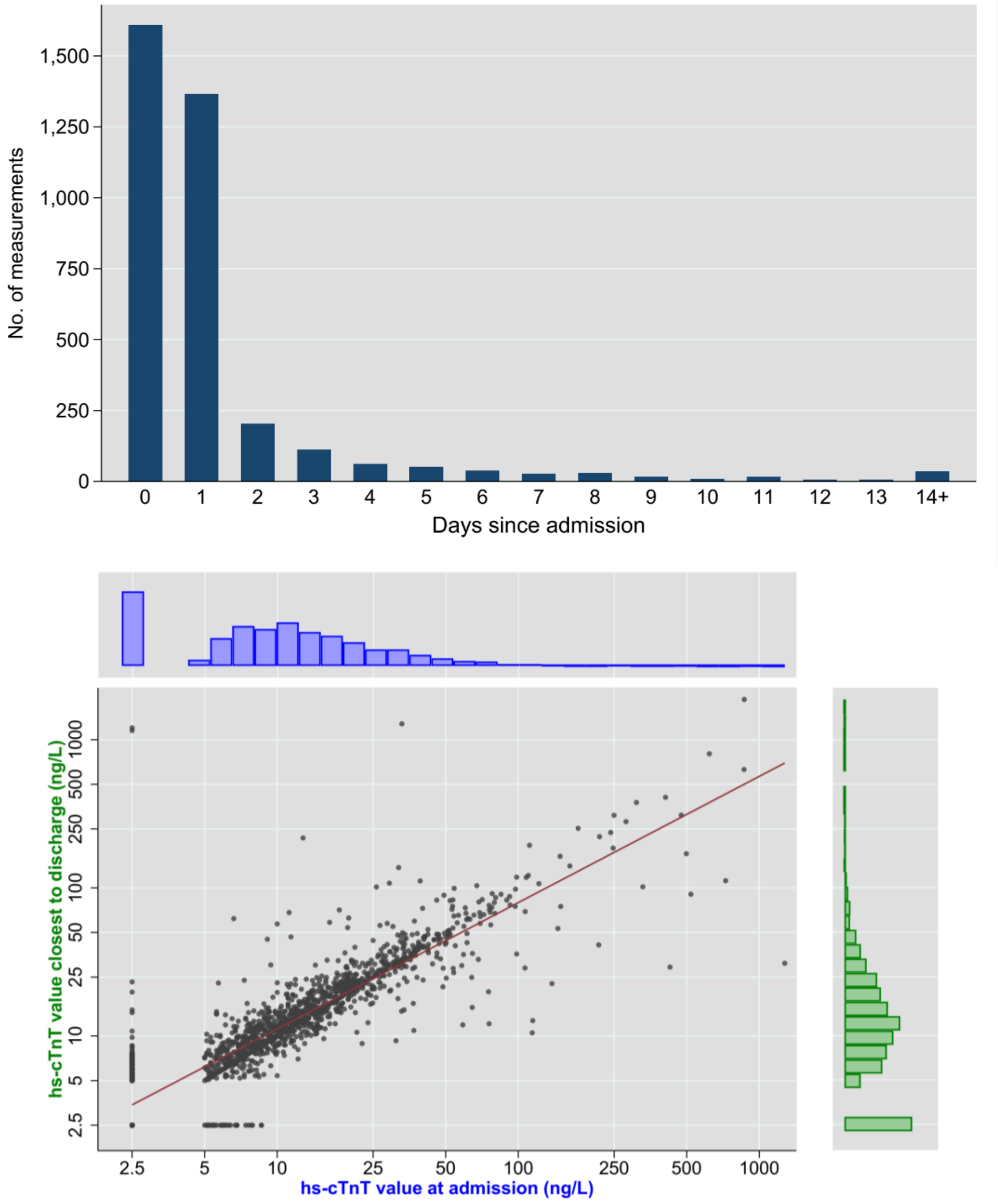

In total, 3588 measurements of hs-cTnt were performed in 1687 patients. The age- and sex-adjusted partial correlation coefficient of log-transformed hs-cTnT values measured at admission vs. closest to discharge was 0.834 (95% CI: 0.819 to 0.848; P<0.001). The histogram with bars in blue color shows the distribution of hs-cTnT values at admission and the histogram with bars in green color shows the distribution of hs-cTnT values closest to discharge. Hs-cTnT = high-sensitivity cardiac troponin T.

SUPPLEMENTAL FIGURE 2 Comparison of the strength of association of hs-cTnT with the combined CVD endpoint to other cardiovascular risk markers.

Variable	Beta	Standard error	z statistic	Hazard ratio for CVD per 1-SD higher or lower[a] level (95% CI)	P value
Log hs-cTnT	0.334	0.099	3.36	1.40 (1.15, 1.70)	<0.001
HDL cholesterol[a]	0.329	0.118	2.79	1.39 (1.10, 1.75)	0.005
Age	0.197	0.130	1.51	1.22 (0.94, 1.57)	0.131
Log hs-CRP	0.167	0.100	1.67	1.18 (0.97, 1.44)	0.095
Systolic blood pressure	0.069	0.095	0.73	1.07 (0.89, 1.29)	0.466
LDL cholesterol	0.008	0.101	0.08	1.01 (0.83, 1.23)	0.938
Log NT-proBNP[b]	0.007	0.137	0.05	1.01 (0.77, 1.32)	0.959
eGFR	-0.078	0.113	-0.69	0.93 (0.74, 1.16)	0.492

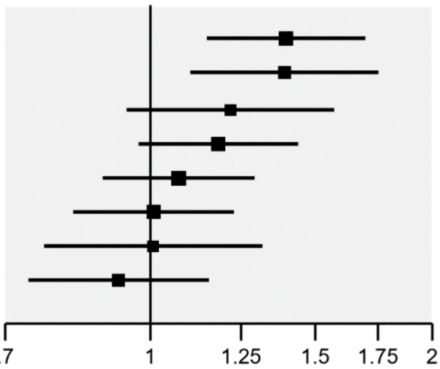

[a]To enable direct comparisons of magnitudes of associations, the hazard ratios presented in this figure are expressed for a 1-standard deviation higher value of each of variables listed herein, except for the hazard ratio for high-density-lipoprotein cholesterol, which is expressed for a 1-standard deviation lower value. [b]Analysis of NT-proBNP involved 1547 patients and 99 CVD events; for head-to-head comparison, the multivariable adjusted hazard ratio of ln hs-cTnT in the same subset of patients was 1.36 (1.07-1.72; P=0.011), the beta coefficient was 0.307 (standard error 0.121) and the z statistic was 2.54. CVD = cardiovascular disease; eGFR = estimated glomerular filtration rate; HDL = high-density-lipoprotein; LDL, low-density-lipoprotein; hs-CRP = high-sensitivity C-reactive protein; hs-cTnT = high-sensitivity cardiac troponin T; NT-proBNP = N-terminal pro-B-type natriuretic peptide.

SUPPLEMENTAL FIGURE 3 Association of hs-cTnT with the risk of the combined CVD endpoint across pre-specified subgroups

Subgroup	No. of events / participants	Multivariable adjusted[a] HR (95% CI) for CVD per 1-SD higher log-transformed hs-cTnT	P value	P value for interaction
**Trial arm**				
Standard care	49 / 559	1.50 (1.16, 1.94)	0.002	0.444
STROKE-CARD care	61 / 1128	1.31 (1.02, 1.69)	0.037	
**Type of qualifying event**				
Ischaemic stroke	92 / 1398	1.33 (1.08, 1.63)	0.007	0.033
TIA	18 / 289	2.73 (1.42, 5.23)	0.002	
**Cardioembolic etiology**				
No	78 / 1250	1.31 (1.03, 1.65)	0.026	0.211
Yes	32 / 437	1.67 (1.20, 2.31)	0.002	
**Age category**				
<70 years	39 / 742	1.28 (0.96, 1.71)	0.098	0.378
≥70 years	71 / 945	1.51 (1.18, 1.93)	0.001	
**Sex**				
Male	69 / 1000	1.48 (1.17, 1.87)	0.001	0.416
Female	41 / 687	1.28 (0.95, 1.72)	0.106	
**Atrial fibrillation**				
No	79 / 1263	1.30 (1.03, 1.64)	0.030	0.155
Yes	31 / 424	1.71 (1.25, 2.35)	<0.001	
**Prior coronary heart disease**				
No	81 / 1433	1.35 (1.05, 1.72)	0.018	0.633
Yes	29 / 254	1.48 (1.10, 1.98)	0.010	
**Prior heart failure**				
No	92 / 1532	1.37 (1.10, 1.70)	0.005	0.655
Yes	18 / 155	1.52 (1.00, 2.30)	0.048	
**National Institutes of Health Stroke Scale**				
5 or less	86 / 1304	1.47 (1.19, 1.82)	<0.001	0.342
>5	24 / 383	1.20 (0.81, 1.77)	0.361	

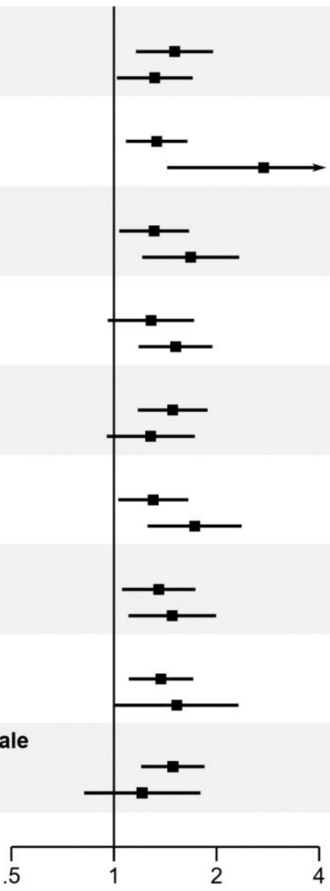

For this subgroup analysis, a Bonferroni-corrected significance level of P<0.0056 was used. [a]Adjusted for age, sex, prior coronary heart disease, prior heart failure, diabetes, smoking, systolic blood pressure, and low- and high-density-lipoprotein cholesterol, unless the variable was used as a subgroup variable. CVD = cardiovascular disease; hs-cTnT = high-sensitivity cardiac troponin T; TIA = transient ischemic attack.
